# Algae-Derived Bioactive Compounds with Anti-Lung Cancer Potential

**DOI:** 10.3390/md18040197

**Published:** 2020-04-08

**Authors:** Imen Saadaoui, Rihab Rasheed, Nabeel Abdulrahman, Touria Bounnit, Maroua Cherif, Hareb Al Jabri, Fatima Mraiche

**Affiliations:** 1 Algal Technologies Program, Center for Sustainable Development, Qatar University, P.O. Box 2713 Doha, Qatar; Rihabrasheed@qu.edu.qa (R.R.); touria.bounnit@qu.edu.qa (T.B.); cherif.maroua@qu.edu.qa (M.C.); h.aljabri@qu.edu.qa (H.A.J.); 2 Translational Research Institute, Academic Health System, Hamad Medical Corporation, P.O. Box 3050 Doha, Qatar; kcnabeel87@gmail.com; 3 Department of Pharmaceutical Sciences, College of Pharmacy, QU Health, Qatar University, P.O. Box 2713 Doha, Qatar; fatima.mraiche@qu.edu.qa

**Keywords:** bioactives, drug discovery, marine algae, mode of action, molecular target, lung cancer

## Abstract

Lung cancer is one of the major causes of death worldwide. Natural molecules with anti-lung cancer potential are of a great interest and considered as very promising alternative to substitute or enhance the efficiency of the conventional drugs. Recently, algae as source of high value-added compounds are considered as very promising source of these bioactive molecules. These are secondary metabolites that consist mainly of derivatives of peptides, carbohydrates, and lipids with various structures. Accordingly, various mechanisms by which different algae molecules demonstrate attenuation of tumor angiogenesis were stated and discussed. The mode of action of the algae bioactives is closely related to their nature and chemical structure. Furthermore, this literature review considers the synergistic effect between microalgae bioactives and conventional drugs and discuss the economic feasibility of producing microalgae bioactives at large scale to conclude with some future perspectives related to algae-based drug discovery.

## 1. Introduction

Cancer is a critical worldwide public health challenge and specifically lung cancer is the leading cause of cancer-related deaths [1,2]. Non-small cell lung cancer (NSCLC) and small cell lung cancer (SCLC) represent the two main types of Lung cancers with NSCLC occurring most commonly with a frequency of 85%. It was previously described that the 5 years survival rate of NSCLC and SCLC is 18% and 6%, respectively [3]. The three major histologic subtypes of NSCLC include adenocarcinomas, squamous cell carcinoma and large cell lung cancer. Adenocarcinoma, accounts for 40% of all lung cancers as is considered as the most common histological variant identified in non-smokers. This cancer type is frequent especially in females less than 60 years old [4]. Unfortunately, conventional treatment approaches including surgery radiotherapy and chemotherapy showed limited success rate.

Therefore, it is essential to identify alternative treatments to regress the development of lung cancer. Several research works are being carried out to discover drugs against cancer from natural origins such as plants and succeeded to identify over 195,000 bioactives with known quantitative interactions [5]. These compounds were tested on clinical level and recent studies done on 453 cancer patients showed that 77% of patients complement the conventional treatment with specific herbal medicines to reduce the symptoms related to cancer by improving the immune system, or even eliminate the cancer directly [6]. As an example, a study done by the cancer national research institute proved that high dose of Vitamin A increases the number of germinal centers to enhance the maturity of the antibodies in tumor and lymph tissues which may be beneficial to patients with NSCLC.

In addition to plants, many widely described chemotherapeutic agents from microorganism such as cyanobacteria and microalgae have been discovered by in vitro assays [3]. Indeed, an estimate of 72,500 algal species have been identified, out of which the majority are of marine origin [7]. Several algae-based compounds, such as polysaccharides, steroids, fatty acids, carotenoids, halogenated compounds, and peptides, have exhibited a large number of biological properties including anticancer activity. These bioactive molecules bind to diverse sites, suppressing cell divisions, or inducing apoptosis via activation of specific cellular pathways [8]. 

Furthermore, previous investigation demonstrated that cancer cell killing efficiency of chemotherapeutic drugs was achieved with a very low dose once combined with Chinese herbs [9]. Furthermore, active compounds from natural sources showing reduced side-effects are of great interest due to their cytotoxic and chemosensitizing activities in addition to their synergistic interactions. 

The increasing interest on using microalgae as natural, safe and renewable source of nutraceuticals and pharmaceuticals to fight cancer has led to rise in the research activities and related publications in this field. However, the focus was on specific health applications and a few numbers of publications was related to anti-lung cancer investigations. The current review article presented for the first time, to the best of our knowledge, a deep description of micro algae high-value compounds active against lung cancer considered as a big health challenge worldwide. Additionally, the mode of action for particular molecules will be described and discussed. Furthermore, this review paper considers the synergistic effect between microalgae bioactives and conventional drugs and conclude with discussing the economic feasibility of producing microalgae bioactives and presenting some future perspectives related to algae-based drug discovery. 

## 2. Molecular Origin and Signaling Pathways Associated to Lung Cancer 

The pathogenesis of human cancer is associated with multiple molecular abnormalities which lead to acquisition of cellular features, including enhanced angiogenesis, replicative immortality, cell death resistance, continuous proliferative signaling, inactivation of growth suppressors, and induction of invasion and metastasis [10]. Lung cancer is induced by genetic and epigenetic alterations including mutations, inhibition of tumor suppressor genes and overexpression of growth promoting oncogenes [11]. Commonly stimulated oncogenes in lung cancer are EGFR, ERBB2, MYC, KRAS, MET, CCND1, CDK4, MET, EML4-ALK fusion, and BCL2 [12]. Among these stimulators, EGFR also known as ErbB-1was the first receptor tyrosine kinase (RTK) identified and the first one linked to cancer. Furthermore, this receptor is the most intensively studied among all RTKs [13]. EGFR is overexpressed in 50–90% of NSCLCs, therefore, tyrosine kinase inhibitors namely, gefitinib and erlotinib, and monoclonal antibody cetuximab were developed to target EGFR signaling [12]. 

Furthermore, the lack of sensitivity of NSCLC cell lines to EGFR inhibitors was associated with persistent activity of ERK or Akt kinase pathways [14]. In addition, mutations of the KRAS oncogene have been suggested to contribute to the lack of sensitivity of EGFR tyrosine kinase inhibitors in NSCLC [15]. Another proposed mechanism that has been suggested to contribute to the resistance of EGFR inhibitors is the amplification of the Met proto-oncogene [16]. Impairment of EGFR tyrosine kinase inhibitors mediated apoptosis pathways (Bcl2 like 11/BIM deletion polymorphism) are other mechanisms that contribute to resistance of EGFR targeted drugs [17].

## 3. Photosynthetic Microorganisms as a Natural and Renewable Source of Anti-Lung Cancer Agents 

Marine flora comprises of brown, blue, green, blue-green and red-algae. Among them, primarily, brown algae were used as a source of secondary metabolites with multiple biological activities. Marine microalgae, phytoplankton, are considered as an important constituent in the marine food chain [18,19]. They are attracting enormous attention and have been producing molecules which are chemically and pharmacologically novel with diverse biological efficacies [20]. These valuable molecules belong to different classes of algae such as microalgae, macroalgae and cyanobacteria and the current literature review focuses on biomolecules that are active against lung cancer.

### 3.1. Macroalgae

Marine macroalgae, designated as seaweeds is most dominant in the marine flora with a frequency of 90%. The seaweeds represent also 50% of the universal photosynthesis quota [21]. Seaweeds have been used in traditional medicine for more than 2000 years in China [22]. Due to their ability to fight numerous diseases such as gall stones, stomach ailments, eczema, cancer, renal disorders, scabies, psoriasis, asthma, arteriosclerosis, heart disease, lung diseases, and ulcers, seaweeds have attracted the attention of the scientific community in the last three decades [23]. The pharmaceutical potential of those molecules was amply investigated for drug discovery. Consequently, several high-value compounds (HVC) were identified like carotenoid, polysaccharides, fatty acids, glycoproteins, haloforms, halogenated alkanes, alkenes, alcohols, aldehydes, hydroquinones, ketones, phlorotannins, pigments, lectins, alkaloids, terpenoids, sterols, and some heterocyclic and phenolic compounds. Many of these HVC are in clinical or preclinical trials. 

### 3.2. Microalgae 

Microalgae are autotrophic microorganisms that consume CO_2_, light, and inorganic nutrients to produce biomass, rich in primary metabolites such as lipids, carbohydrates, proteins, and pigments. In addition, microalgae can produce HVC such as polysaccharides, poly-unsaturated fatty acids (PUFAs), carotenoids (lutein, zeaxanthin, and astaxanthin), and vitamins with very high nutraceutical and pharmaceutical potentials [24]. For example, *Tetraselmis suecica*, a marine green microalgae belonging to the class Chlorophyceae, is enriched with biomolecules with an amount of 74 g of PUFAs per kg of microalgae harvested [25]. Its crude extract showed strong antioxidant and cell repairing activity in vitro in the human lung adenocarcinoma cell line A549. It has also been demonstrated that extract from a heterogeneous mixture of microalgae inhibited the colony forming ability of A549 and H460 lung cancer cells at a dose of 5 µg µL^−1^ [26]. Moreover, the proliferation and cell migration of H1299, A549, and H1437 lung cancer cells were markedly inhibited by *Chlorella vulgaris* extract with a dose of 200 µg mL^−1^ [27]. Varying the polarity of the solvents used for the extraction will help to dissolve and extract most of the biomolecules that are selectively active against specific type of cancer cells. Ethanolic extracts of *H. musciformis*, *P. gymnospora*, and *D. dichotoma* showed selectivity towards NCI-H292 cells (human lung mucoepidermoid carcinoma) 22.0 ± 3.5 μg mL^−1^, whereas, the dichloromethane extract, chloroform extract and methanolic extracts of *D. dichotoma* were active against HEp-2 (human larynx epidermoid carcinoma) cells [28].

### 3.3. Cyanobacteria

Blue green algae (cyanobacteria), a cyanophyta, possess different mechanisms to produce various cyclic nitrogenous compounds that have potent biological activities. For example, *Lyngbya majuscula* produces several molecules such as obyanamide, hectochlorin, lyngbyastatin 3, and apratoxin with proven cytotoxic activities [20]. Similarly, *Nostoc* species are also known to generate compounds like cryptophycin whose analogues are very effective against cancer (Hela cells) [29]. Deniz et al [30], emphasized the use of phycocyanins from *Spirulina platensis* against A549 lung cancer cell line with an IC50 value of 29.41 μg mL^−1^ after 24 h of incubation [30,31]. 

## 4. Bioactive Compounds from Algae 

HVC in algae differ in terms of their chemical properties. Phenolics and carotenoids are the most studied microalgal phytochemicals. Guedes et al [28] showed a selective activity of algae extracts towards NCI-H292 lung cancer cells [28]. Ethanol extracts of *H. musciformis* and *P. gymnospora* presented anti-proliferative activity with IC50 of 22.0 ± 3.5 μg mL^−1^ and 15.9 ± 2.8 μg mL^−1^, respectively. However, for *Dictyota dichotoma*, the anticancer capacity was observed using both ethanol and chloroform extracts with IC50 of 25.2 ± 1.1 μg mL^−1^ and 22.0 ± 3.5 μg mL^−1^, respectively. Several chemical components were identified as active against lung cancer such as: Polysaccharides, sulfate polysaccharides, terpenoids, and peptides. Table 1 and Table 2 group several examples of macroalgae and microalgae bioactives respectively, with their origins, targets and mode of action. All anticancer investigations should be performed using the American Cancer Institute (NCI) protocol, which considers a plant crude extract and pure substances as interesting if it showed IC50 less than 30 μg mL^−1^ and 4 μg mL^−1^, respectively [32].

### 4.1. Derivatives of Carbohydrates 

#### 4.1.1. Polysaccharides 

**Figure 1:**
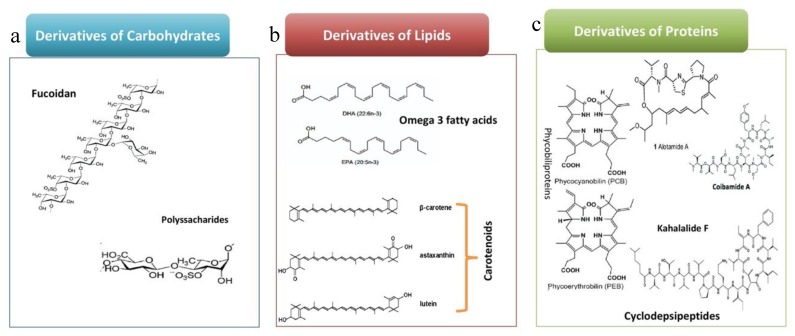
Chemical Structure of the algae bioactives with against lung cancer potential (**a**) derivatives of carbohydrates; (**b**) derivatives of lipids; and (**c**) derivatives of proteins.

Polysaccharides (PS) are polymers of simple sugar (monosaccharide) allied by glycosidic bonds (Figure 1). They are considered as the major algae component (reaching up to 76% of the dry weight) since they play a key role in the cell wall structure as well as in physiological functions. Several marine algae PS were subjected for in vitro and in vivo investigations for their anticancer potential [55,56]. The PS bioactivity is closely dependent on their physicochemical properties [36]. These properties depend strongly on the nature of the algae that produces it. Kang et al [36] succeeded recently to identify a neutral polysaccharide from *Gracilariopsis lemaneiformis* with a linear structure of repeated disaccharide agarobiose units consisting of 3,6-anhydro-L-galactose and D-galactose. Such polysaccharides showed high activity against A549 lung cancer cell line (Table 1). 

#### 4.1.2. Fucoidan 

Fucoidans are sulfated polysaccharides generally produced by brown algae such as: *Sargassum thunbergi* [57], *Ascophyllum nodosum* [58], *Viz fucusvesiculosus* [59], *Laminaria japonica* [60], *Fucus evanescens*, and *Laminaria cichorioides* [61]. It was stated that algae fucoidans present high anticancer activity against several cancer types, including lung cancer, via targeting the key apoptotic molecules. Previous study demonstrated that fucoidans inhibit lung cancer through Smurf2 dependent ubiquitin degradation of TGFβ receptors [36]. It was also reported that prophylactic administration of fucoidans suppress lung cancer metastasis by inhibiting MMPs and VEGF [57]. Besides that, fucoidans have the ability to present a synergistic effect towards the anticancer agents currently in use [41]. This has emphasized the need for further research by combining such polysaccharides with the existing medicines to improve the efficacy of conventional drugs. Atashrazm et al [62] evidenced also its beneficial effects as polysaccharides can prevent from the toxic effect associated to the conventional therapies. The chemical structure of the fucoidan is present in the Figure 1.

### 4.2. Derivatives of Protein

#### 4.2.1. Phycobiliprotein 

Phycobiliproteins are composed of protein covalently linked to chromophore called phycobilins (Figure 1). Accordingly, they are considered as strong fluorescent markers. These proteins are water soluble and present antioxidant properties [49,63]. Phycocyanin and phycoerythrin are the most widely known phycobiliproteins that have been produced commercially to be used as natural food colorant. Phycocyanin which is a blue colored phycobiliprotein produced essentially from *Arthrospira* sp., reportedly showed anticancer properties against A549 lung cancer cells [30]. Previous study mentioned the use of phycocyanin individually and/or in combination with other agents to obtain anticancer effects against lung cancer [40,41]. Phycoerythrin (pink colored protein pigment) produced by a marine *Lyngbya* showed apoptotic activity against A549 human lung carcinoma cells [64].

#### 4.2.2. Glycoprotein 

Glycoprotein consists of proteins bound to carbohydrates. Very recently, Senthilkumar and Jayanthi [40] successfully isolated, purified and characterized a glycoprotein of size 48KDa from *Codium decorticatum* using HPLC, IR, NMR, and Circular Dichroism (CD). Carbohydrates representing 36.24% of the glycoprotein consist of: Rhamnose, galactose, glucose, and mannose with a mole ratio of 38:30:26:6. Using FT-IR and NMR spectra, the authors proved that sugars are attached to the protein via (1→4)-linked β-galactose residues and β-linked glucose residues. The investigation of the anticancer activity of this glycoprotein expressed interesting activity against A549 lung cancer cells with IC50 of ~40 g mL^−1^ after 48 h of incubation. Such activity is time and dose dependent. Using fluorescence microscopy, these authors proved that glycoproteins induce apoptosis.

#### 4.2.3. The Cyclo Depsipeptides

- Kahalalides F

The Kahalalides consist of series of depsipeptide isolated from *Bryopsis pennata* [65]. Among them, the Kahalalide F, which contains 13 amino acids and 5-methylhexanoic acid at its N-terminus, was described as highly active against solid tumors including lung cancer (Figure 1). A significant in vitro activity was recorded against human non-small cell lung cancer cells (A549) with an IC50 of 2.5 µg mL^−1^ [65]. Furthermore, patient treated ex vivo with 0.01–1 µM of Kahalalide F showed a total inhibition of the non-small-cell lung cancer. The investigation of chemical properties of this molecule for drug discovery currently reached phase II trials by PharmaMar [66]. The stereochemistry of the Kahalalide F was assessed by several researchers but only through total synthesis that Lopez-Macia et al. [67] succeeded to firmly identify it and suggested that the cyclized and linear side chains play a critical role in the biological activity of the drug [65].

- Other cyclo depsipeptide from cyanobacteria

Several cyclo depsipeptides, chains of 50 or less amino acids, have been isolated from blue green algae such as Coibamide A, Alotmide A, Veraguamide A [68]. Coibamide A was isolated from cyanobacterium *Leptolyngbya sp*. The chemical structure was determined using NMR and mass spectroscopy. The excessive number of N and O methylation and the abundance of alkyl amino constituents make the structure very complicated. Such molecule showed cytotoxicity against NCIH- 460 lung cancer cells. Alotmide A, a cyclic depsipeptide isolated from *Lyngbya majuscula* and *Lyngbya sordida* showed cytotoxicity against H-460 human lung cancer cells [51]. Similar action was exhibited by Palmyramide A and Dolastatin 16 on the same cell line [69]. Finally, Veraguamide A, a cyclic peptide from *Oscillatoria margaritifera* showed cytotoxicity against cancer cells with LD50 equal to 141 nM [70].

### 4.3. Derivatives of Lipids 

#### 4.3.1. Carotenoids 

Carotenoids are the most diverse and widespread lipophilic colored compounds in nature [71,72]. The major carotenoids found in microalgae are astaxanthin, β-carotene, lutein, lycopene, and canthaxanthin (Figure 1). Carotenoids can be over produced in algae culture by exposing it to strong or continuous light from 50 to over 1250 μmol photon m^−2^ s^−1^ [73]. High temperatures and modification of the growth media favors their production [74]. Zhang et al [42] modeled that light attenuation, temperature, and lower nitrogen sources were optimal for astaxanthin production. Similarly, lycopene inhibited human lung cancer cells (NCI-H226) proliferation and suppressed insulin-like growth factor-I stimulated growth which are the major autocrine/paracrine regulators of mammary and endometrial cancer cell growth [43]. Analogously, astaxanthin, produced commonly by *Haematococcus* sp. [30], a non-provitamin A carotenoid, has recently gained attention by inhibiting cell viability and proliferation of two NSCLC, A549 and H1703 [75]. Hence, microalgal carotenoids possess substantial potential against lung carcinomas. 

#### 4.3.2. Omega Fatty Acids 

Fatty acids are a group of secondary metabolites synthesized by Claisen condensation reactions between acetyl-CoA and malonyl-CoA with the action of fatty acid synthases. Marine microalgae such as *Isochrysis*, *Tetraselmis*, *Chaetoceros*, *Thalassiosira*, and *Nannochloropsis* are known with high ability to produce polyunsaturated fatty acids (PUFAs) with multiple health benefits [76,77]. These molecules exert anticancer actions against lung and gastrointestinal carcinomas [78]. Its implication in lung cancer is shown by in vivo studies demonstrating slow growth of lung cancer in mice when fed with eicosapentaenoic acid (EPA) or docosahexaenoic acid (DHA) rich food [47,79]. The anti-proliferative effect of DHA and EPA on A549 lung cancer cell lines was confirmed by different in vitro assays suggesting their potential therapeutic role [80]. 

### 4.4. Tuberatolide B (TTB, C27H34O4) 

Tuberatolide B was isolated for the first time from the Korean *Sargassum macrocarpum* and identified as diastereomeric meroterpenoid that acts as a Farnesoid X receptor (FXR) antagonist [81]. Recently, Choi et al [38] proved that TTB induced the ROS production leading to STAT3 inhibition, DNA damage, and apoptotic cell death. Therefore, TTB suppresses cancer progression by promoting ROS-mediated inhibition of STAT3 signaling. 

## 5. Anticancer Mode of Action and Putative Targets 

Knowledge of mechanism by which various molecules exert their effects is important to design drugs for treating cancer and to improve the lifestyle of those at risks. There are various mechanisms by which different molecules demonstrate attenuation of tumor angiogenesis [82], promotion of cell cycle arrest [83,84], induction of apoptosis [36,85], or necrosis and immuno stimulation [86]. The mechanism by which algae bioactives function is mainly based on the nature and chemical properties of the algae bioactives. Atashrazm et al [62] stated that fucoidan fights lung cancer via delaying cancer development, eliminating cancer cells, and presenting synergistic effect with the anticancer chemotherapeutic agents, simultaneously.

Algae bioactives trigger apoptosis via several pathways. Very recently, Kang et al [36] proved that *Gracilariopsis lemaneiformis* PS stimulates apoptosis via Fas/FasL pathway. However, the *Sargassum fusiforme* PS decreased the expression of CD31, VEGF-A and tumor microvessel density [37]. In addition, it inhibits the expression of VEGF-A in tumor cells and its receptor VEGFR2 in human umbilical vein endothelial cells. Accordingly, PS of *S. fusiforme* are considered an alternative anti-lung cancer drug.

Choi et al. [38] proved that tuberatolide B inhibits lung cancer growth via the histone H2AX protein, which is important in the DNA damage response (DDR) pathway. The formation of *γ*H2AX foci on double stranded DNA activates the DDR pathway. Tuberatolide enhances DNA damage by inducing γH2AX foci formation and the phosphorylation of DNA damage-related proteins such as Chk2 and H2AX. Furthermore, Tuberatolide decreases the expression of BCL2 and increases the cleavage of caspase 3 and PARP and increases the percentage of annexin V-positive apoptotic cells. An important determinant of cellular response to DNA damage is the level of intracellular reactive oxygen species (ROS); Tuberatolide B induces the formation of ROS, which could act as a cellular toxicant. 

**Figure 2:**
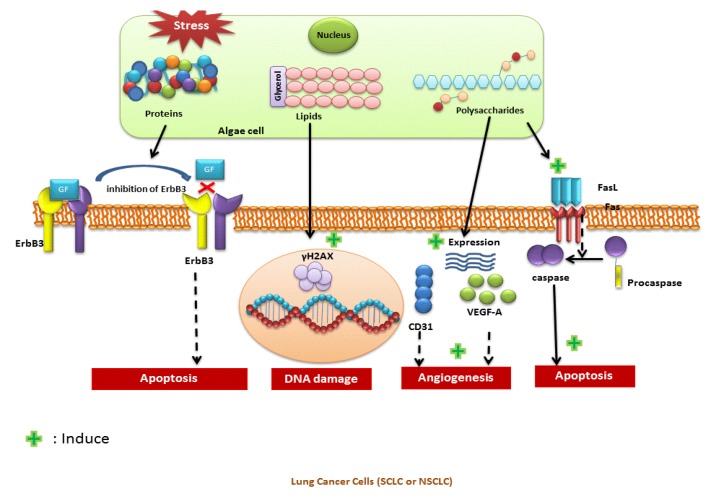
Examples of Mode of action of the major photosynthetic bioactives against lung cancer.

Although significant advances have been made during the last 24 years in the purification, identification and biological and chemical characterization of depsipeptide, their mode of action is not completely understood. The putative mode of action of the microalgae bioactives is presented in the Figure 2. It was stated that PI3K/Akt signaling pathway coupled to ErbB3 receptors could be the target [87]. There are no clinical reports related to potential ErbB3 inhibiting drugs, Accordingly, Kahalalide F would be considered as a promising candidate for inhibition of ErbB3 receptors in tumor cells [88].

Several postulates have been made to explain the action of omegas against cancer cells. Apoptosis, the process of programmed cell death is controlled by caspases [89]. EPA and DHA are known to increase caspases specifically 3 and 9, that induce apoptosis of cancer cells [90]. It is reported that DHA stimulates autophagy via p53-mediated AMPK/mTOR signaling [91]. Auto phagosomes have been induced upon treatment of A549 cells with DHA or EPA. Furthermore, it was stated that DHA reduced the expression of COX-2 and suppressed the formation of pro inflammatory lipid mediator, prostaglandin E2, leading to anti-proliferative effect. It was ultimately proved that omega3 fatty acids reduced the COX2 expression by downregulating NF-κB [92]. Additionally, several other factors such as: Ras transcription factors and protein kinases were considered as potential targets of omega3 fatty acids mediated tumor inhibition [78,93].

The anticancer activity of carotenoid derivatives is mediated by the activation of several receptors and augmentation of carcinogen-metabolizing enzymes [94]. Whereas, phycocyanin can inhibit the proliferation of the tumor cells via arrest cell cycle at the G0/G1 phase and block DNA synthesis [95]. It was seen that phycocyanin molecules triggered apoptosis by targeting cellular proteins via intrinsic and extrinsic pathways. Several molecules responsible of inducing apoptosis such as: CDK-4, TNF, caspase-3, were identified as molecular targets of phycocyanin [96].

## 6. Potential to Combine Natural Products with Existing Drug Regimens in the Treatment of Lung Cancer

The use of natural products in combination with conventional chemotherapeutic agents like cisplatin has the potential to enhance the therapeutic effectiveness of common chemotherapy agents through cancer. Hence, the synergistic enhancement of cisplatin therapy would be a beneficial strategy to overcome the severe toxic side effects and drug resistance of cisplatin. In line with such concept [97], some of the compounds isolated from algae can also be used as co-adjuvants to improve the efficiency of the drugs currently used as therapeutics. Indeed, the pre-treatment of HepG2 cells with fucoxanthin allowed to improve the therapeutic effect of cisplatin [98]. Additionally, the conjugation of λ-carrageenan [99], Cf-PLS [100], and porphyran [101] compounds with 5-FU drug enhanced its antitumor activity. Furthermore, Psammaplin A, a phenolic compound containing a sulfur bridge accruing naturally in monomers or dimers, is produced by microalgae and cyanobacteria. Charkie [102] demonstrated that, can enhance the activity of camptothecin (a DNA damage inducing drug) against different kind of cancers including Bap1-null lung cancer via inhibiting the histone deacetylase (HDAC) responsible of tumourigenesis and angiogenesis.

## 7. Economic Feasibility and Challenges in Using Microalgae for Lung Cancer

It is evident from our review that microalgae have high potential as a source of anti-lung cancer molecules, however it still needs to be extensively investigated further, in vitro and in vivo [103]. Although microalgae present high potential for producing pharmaceuticals and nutraceuticals with high antioxidant and anticancer activities, some limitations and challenges need to be addressed carefully especially before moving from laboratory to pilot and industrial scale [104]. Some challenges are related to the upstream part leading the biomass production and here we present as example the selection of the most suitable cultivation system for producing biomass enriched with high value products and contamination free. This normally requires the use of photobioreactors which present high cost comparatively to the open raceway pond which is considered as the most common and economically feasible way to produce microalgae biomass. On the other hand, studies have proven that the production of HVC from algae can be enhanced by various physicochemical stresses [105,106,107]. Thus, the optimization of the best conditions for producing microalgae biomass enriched with high-value products requires more energy and cost [108]. 

Additionally, the downstream processing of the microalgae biomass to extract the high-value products is still unviable and needs a lot of optimizations of the different steps. New low-cost harvesting technology is required to make the whole process sustainable since harvesting is considered as a major challenge as it is requiring almost 30% of the total cost of the pharmaceuticals production process. Different initiatives are recently developed but still they didn’t meet the viability [109,110,111,112]. Furthermore, the extraction process that is efficient, safe for the molecules, economically feasible and environmentally friendly is far from being a reality. For that, a lot of optimizations are urgently required. Some initiatives targeting a cost-effective process to extract the microalgae bioactives are also existing but still they did not meet the sustainability required [113]. Finally, microalgae bioactives are very promising as pharmaceuticals and efficient drugs against lung cancer however a lot of optimizations are required to make the up and down stream processing steps viable and economically feasible. For the time being, including microalgae biomass enriched with high value products in the human diets could be a very good option to enhance the immune system and improve the human health.

## 8. Future Perspective

Cancer is a serious problem constituting the second most threatening disease after cardiac complications [114]. Microalgae emerged as a vast, and largely untapped potential repository of natural compounds. It is not only used as functional foods but also have a long history of use in Asian countries in treatment of cancer. Many crude or partially purified molecules especially polysaccharides have been tested for their antitumor activities. 

Different Genus such as *Chlorella*, *Cladophoropsis*, *Codium*, *Dunaliella*, *Enteromorpha*, *Helimenda*, *Udocea,* and *Ulva* were previously reported to be able to produce HVC which exhibit Bioactive properties against cancer [40,43,44,46,114]. Strains isolated from desert harsh environment are poorly investigated for their potential repository of anticancerous natural compounds. Hence, further examination of local isolated algal species is necessary to truly investigate this great resource and highlight the importance of using this natural molecule to complement the existing medicines and provide similar results with lesser or no side effects. In the future, with achieving a viable and economically feasible production of marine microalga biomass, microalgae bioactives will represent the most promising natural alternative for chemical drugs with higher recovery rates and lesser side effects. 

## Figures and Tables

**Table 1:** Macroalgae bioactive molecules with biological source and activity against lung cancer cells.

Algae Species	Active Molecule	Lung Cancer Cells	Dose IC50	Inhibition Pathways	Ref.
*U. pinnatifid* algae	Fucoidan	Human lung cancer A549 cells	50, 100, 200 μg mL^−1^	↓ procaspase 3 PARP cleavage Caspase-9 activation ↓ procaspase 3 ↓ Bcl-2, ↑ Bax	[33]
*F. vesiculosus*	Fucoidan	lung cancer A549 cells	50–400 μg mL^−1^	inhibited the MMP-2 and MMP-9 protein expression, cell migration, and invasion activities of LLC cells	[34]
*Bryopsis*	Kahalalide F	lung cancer A549 cells	2.5 µg L^−1^	Targets the lysosomes,	[35]
*Hypnea musciformis*	Chloroform extract	NCI-H292 (human lung mucoepidermoid carcinoma)	15.0 ± 1.3 μg mL^−1^	-	[28]
	Ethanol extract	NCI-H292	22.0 ± 3.5 μg mL^−1^	-	[28]
*Gracilariopsis lemaneiformis*	Polysaccharide	NSCLC cell line, A549	50 μg mL^−1^	-	[36]
*Sargassum fusiforme*	Ploysaccharide	lung adenocarcinoma SPC-A-1 cells and its xenograft model	-	inhibit VEGF-A-related angiogenesis and proliferation	[37]
*Sargassum macrocarpum*	Tuberatolide B (TTB, C27H34O4)	(A549 and H1299)	-	promotes ROS, mediated inhibition of STAT3 signaling, decreased the expression of Bcl2, increases the cleavage of caspase-3 and PARP, enhances the percentage of annexin V, positive apoptotic cells, induces ROS generation, induces DNA Damage, induces the phosphorylation of Chk2 and H2AX.	[38]
*Plocamium cartilagineum*	halogenated monoterpene 1 (5E,7Z)-3 48-trichloro-7-dichloromethyl-3-methyl-157-octatriene)	(NCI-H460)	4 μg mL^−1^	-	[39]
*Codium decorticatum*	glycoprotein	A549 lung cancer	40 ± 0.41 μg mL^−1^	Induction of apoptosis	[40]
*Gracilaria edulis*	phytol, a diterpene	A549 lung cancer	24.5 ± 19.1 μg mL^−1^ at 48 h	-	[41]

-: not determined; Ref.: Reference; ROS: Reactive oxygen species.

**Table 2:** Microalgae bioactive molecules with biological source and activity against lung cancer cells.

Algae Species	Active Molecule	Lung Cancer Cells	Dose IC50	Inhibition Pathways	Ref.
*Haematococcus* sp.	Astaxanthin	NSCLC	5–25 μg mL^−1^	↓ MKK1/2-ERK1/2 inducing cytotoxicity against cancer	[42]
*Chlorella zofingiensis*	Lycopene	A549	3–5 mM	↓ mRNA and protein levels of cyclin E	[43,44,45]
*Dunaliella salina*	Beta-carotene	A549	25–100 μg mL^−1^	↓cell proliferation, induce apoptosis and cell cycle G0/G1 arrest	[46]
*Tetraselmis; Nannochloropsis*	EPA and DHA	A549 H1299	EPA = 6.05 μM 50% inhibition	Generate PGE3 through COX-2 enzyme ↓ Of Akt phosphorylation by PGE3.	[47,48]
*Chlorella Vulgaris*	Polyphenols, Flavonoid	H1299, A549, and H1437	13.40 and 0.46 (μg Gallic acid/g lyophilized extract), 3.18 μg quercetin/g lyophilized extract	Affects migration of cells, inhibits metastasis	[27]
*Lyngbya* sp.	Phycoerythrin	A549	100–200 µg mL^−1^	Cell arrest at G0/G1 phase, ↓ cell viability and mitochondrial membrane potential, an increment in lactate dehydrogenase release	[49]
*Arthrospira platensis, Oscillatoria tenuise*	Phycocyanin	A549	26.82 μg mL^−1^	Cell apoptosis/blebbing necrosis,	[30,50]
*Lyngbya majuscule*, *Lyngbya sordida*	Alotmide A, Aurilide	H-460 NCI–H460	-	Cell apoptosis	[51]
Leptolyngbya	Coibamide	NCI-H460	LC50 < 23 nM	Inhibits cell proliferation through novel mechanism	[52]
*Caldora penicillata*	Laucysteinamide A, Curacin.	H-460	11 uM,	Anti-tubulin formation	[42]
*Gymnodinium*	GA3P, d-galactan sulfate	NCI-H23	2.8	Inhibits topo I and topo II	[53]
		NCI-H226	2.2		
		NCI-H522	1.3		
		NCI-H460	3.8		
		A54	11		
		DMS273	2.0		
		DMS11	2.7		
*P. tricornutum*	Nonyl 8-acetoxy-6-methyloctanoate (NAMO)	A549	35% inhibition at 50 μg mL^−1^	p53 and caspace-3 mediated cell apoptotic pathway	[54]

-: Not Determined; Ref.: Reference.

## References

[1] International Agency for Research on Cancer, WHO World Cancer Report 2014. http://publichealthwell.ie/node/725845.

[2] Curran W.J. (2003). Evolving chemoradiation treatment strategies for locally advanced non-small-cell lung cancer. Oncology.

[3] Cardozo K.H.M., Guaratini T., Barros M.P., Falcão V.R., Tonon A.P., Lopes N.P., Campos S., Torres M.A., Souza A.O., Colepicolo P. (2007). Metabolites from algae with economical impact. Comp. Biochem. Physiol. C Toxicol. Pharmacol..

[4] Samet J.M., Avila-Tang E., Boffetta P., Hannan L.M., Olivo-Marston S., Thun M.J., Rudin C.M. (2009). Lung cancer in never smokers: Clinical epidemiology and environmental risk factors. Clin. Cancer Res..

[5] Banerjee P., Erehman J., Gohlke B.O., Wilhelm T., Preissner R., Dunkel M. (2015). Super Natural II-a database of natural products. Nucleic Acids Res..

[6] Richardson M.A., Sanders T., Palmer J.L., Greisinger A., Singletary S.E. (2000). Complementary/alternative medicine use in a comprehensive cancer center and the implications for oncology. J. Clin. Oncol..

[7] Guiry M.D. (2012). How many species of algae are there?. J. Phycol..

[8] Blunt J.W., Copp B.R., Keyzers R.A., Munro M.H.G., Prinsep M.R. (2015). Marine natural products. Nat. Prod. Rep..

[9] Tseng C.Y., Lin C.H., Wu L.Y., Wang J.S., Chung M.C., Chang J.F., Chao M.W. (2016). Potential combinational anticancer therapy in non-small cell lung cancer with traditional Chinese medicine Sun-Bai-Pi extract and cisplatin. PLoS ONE.

[10] Hanahan D., Weinberg R.A. (2011). Review Hallmarks of Cancer: The Next Generation. Cell.

[11] Panov S.Z. (2005). Molecular biology of the lung cancer. Radiol. Oncol..

[12] Larsen J.E., Minna J.D. (2011). Molecular Biology of Lung Cancer: Clinical Implications. Clin. Chest Med..

[13] Wang Z. (2017). ErbB receptors and cancer. Methods Mol. Biol..

[14] Janmaat M.L., Kruyt F.A.E., Rodriguez J.A., Giaccone G. (2003). Response to epidermal growth factor receptor inhibitors in non-small cell lung cancer cells: Limited antiproliferative effects and absence of apoptosis associated with persistent activity of extracellular signal-regulated kinase or Akt kinase pathways. Clin. Cancer Res..

[15] Gridelli C., Bareschino M.A., Schettino C., Rossi A., Maione P., Ciardiello F. (2007). Erlotinib in non-small cell lung cancer treatment: Current status and future development. Oncologist.

[16] Engelman J.A., Zejnullahu K., Mitsudomi T., Song Y., Hyland C., Joon O.P., Lindeman N., Gale C.M., Zhao X., Christensen J. (2007). MET amplification leads to gefitinib resistance in lung cancer by activating ERBB3 signaling. Science.

[17] Morgillo F., Della Corte C.M., Fasano M., Ciardiello F. (2016). Mechanisms of resistance to EGFR-targeted drugs: Lung cancer. ESMO Open.

[18] Iwamoto C., Minoura K., Hagishita S., Nomoto K., Numata A. (1998). Penostatins F–I, novel cytotoxic metabolites from a Penicillium species separated from an Enteromorpha marine alga. J. Chem. Soc. Perkin Trans..

[19] Iwamoto C., Minoura K., Oka T., Ohta T., Hagishita S., Numata A. (1999). Absolute stereostructures of novel cytotoxic metabolites, penostatins A-E, from a Penicillium species separated from an Enteromorpha alga. Tetrahedron.

[20] Shimizu Y. (1996). Microalgal metabolites: A new perspective. Annu. Rev. Microbiol..

[21] Dhargalkar V.K., Pereira N. (2005). Seaweed: Promising Plant of the Millennium. Sci. Cult..

[22] Chengkui Z., Tseng C.K., Junfu Z., Chang C.F. (1984). Chinese seaweeds in herbal *Source* medicine. Hydrobiologia.

[23] Nabti E. (2017). Biotechnological Applications of Seaweeds.

[24] Markou G., Nerantzis E. (2013). Microalgae for high-value compounds and biofuels production: A review with focus on cultivation under stress conditions. Biotechnol. Adv..

[25] Pérez-López P., González-García S., Ulloa R.G., Sineiro J., Feijoo G., Moreira M.T. (2014). Life cycle assessment of the production of bioactive compounds from Tetraselmis suecica at pilot scale. J. Clean. Prod..

[26] Somasekharan S.P., El-Naggar A., Sorensen P.H., Wang Y., Cheng H. (2016). An Aqueous Extract of Marine Microalgae Exhibits Antimetastatic Activity through Preferential Killing of Suspended Cancer Cells and Anticolony Forming Activity. Evid. Based Complement Altern. Med..

[27] Wang H.M., Pan J.L., Chen C.Y., Chiu C.C., Yang M.H., Chang H.W., Chang J.S. (2010). Identification of anti-lung cancer extract from *Chlorella vulgaris* C-C by antioxidant property using supercritical carbon dioxide extraction. Process Biochem..

[28] Guedes É.A.C., da Silva T.G., Aguiar J.S., de Barros L.D., Pinotti L.M., Sant’Ana A.E.G. (2013). Cytotoxic activity of marine algae against cancerous cells. Rev. Bras. Farmacogn..

[29] Smith C.D., Zhang X., Mooberry S.L., Patterson G.M.L., Moore R.E. (1994). Cryptophycin: A New Antimicrotubule Agent Active against Drug-resistant Cells. Cancer Res..

[30] Deniz I., Ozen M.O., Yesil-Celiktas O. (2016). Supercritical fluid extraction of phycocyanin and investigation of cytotoxicity on human lung cancer cells. J. Supercrit. Fluids.

[31] Tan L.T. (2007). Bioactive natural products from marine cyanobacteria for drug discovery. Phytochemistry.

[32] Cancer Chemotherapy National Service Center (1962). Protocols for screening chemical agents and natural products against animal tumors and other biological systems. Cancer Chemother. Rep..

[33] Boo H.-J., Hyun J.-H., Kim S.-C., Kang J.-I., Kim M.-K., Kim S.-Y., Cho H., Yoo E.-S., Kang H.-K. (2011). Fucoidan from *Undaria pinnatifida* induces apoptosis in A549 human lung carcinoma cells. Phytother. Res..

[34] Huang T.H., Chiu Y.H., Chan Y.L., Chiu Y.H., Wang H., Huang K.C., Li T.L., Hsu K.H., Wu C.J. (2015). Prophylactic administration of fucoidan represses cancer metastasis by inhibiting vascular endothelial growth factor (VEGF) and matrix metalloproteinases (MMPs) in Lewis tumor-bearing mice. Mar. Drugs.

[35] Smit A.J. (2004). Medicinal and pharmaceutical uses of seaweed natural products: A review. J. Appl. Phycol..

[36] Kang Y., Wang Z.J., Xie D., Sun X., Yang W., Zhao X., Xu N. (2017). Characterization and potential antitumor activity of polysaccharide from *gracilariopsis lemaneiformis*. Mar. Drugs.

[37] Chen H., Zhang L., Long X., Li P., Chen S., Kuang W., Guo J. (2017). *Sargassum fusiforme* polysaccharides inhibit VEGF-A-related angiogenesis and proliferation of lung cancer in vitro and in vivo. Biomed. Pharmacother..

[38] Choi Y.K., Kim J., Lee K.M., Choi Y.J., Ye B.R., Kim M.S., Ko S.G., Lee S.H., Kang D.H., Heo S.J. (2017). Tuberatolide B suppresses cancer progression by promoting ROS-mediated inhibition of STAT3 signaling. Mar. Drugs.

[39] Sabry O.M.M., Goeger D.E., Valeriote F.A., Gerwick W.H. (2017). Cytotoxic halogenated monoterpenes from *Plocamium cartilagineum*. Nat. Prod. Res..

[40] Senthilkumar D., Jayanthi S. (2016). Partial characterization and anticancer activities of purified glycoprotein extracted from green seaweed *Codium decorticatum*. J. Funct. Foods.

[41] Sakthivel R., Muniasamy S., Archunan G., Devi K.P. (2016). *Gracilaria edulis* exhibit antiproliferative activity against human lung adenocarcinoma cell line A549 without causing adverse toxic effect in vitro and in vivo. Food Funct..

[42] Zhang D., Wan M., del Rio-Chanona E.A., Huang J., Wang W., Li Y., Vassiliadis V.S. (2016). Dynamic modelling of *Haematococcus pluvialis* photoinduction for astaxanthin production in both attached and suspended photobioreactors. Algal Res..

[43] Levy J., Bosin E., Feldman B., Giat Y., Miinster A., Danilenko M., Sharoni Y. (1995). Lycopene is a more potent inhibitor of human cancer cell proliferation than either alpha-carotene or beta-carotene. Nutr. Cancer.

[44] Cordero B.F., Couso I., Leon R., Rodriguez H., Vargas M.A. (2012). Isolation and characterization of a lycopene ε-cyclase gene of *Chlorella* (*Chromochloris*) *zofingiensis*. Regulation of the carotenogenic pathway by nitrogen and light. Mar. Drugs.

[45] Mein J.R., Lian F., Wang X.D. (2008). Biological activity of lycopene metabolites: Implications for cancer prevention. Nutr. Rev..

[46] Sheu M.-J., Huang G.-J., Wu C.-H., Chen J.-S., Chang H.-Y., Chang S.-J., Chung J.-G. (2008). Ethanol extract of *Dunaliella salina* induces cell cycle arrest and apoptosis in A549 human non-small cell lung cancer cells. In Vivo.

[47] Yam D., Peled A., Shinitzky M. (2001). Suppression of tumor growth and metastasis by dietary fish oil combined with vitamins E and C and cisplatin. Cancer Chemother. Pharmacol..

[48] Yang X., Xie P., Yu Y., Shen H., Deng X., Ma Z., Wang P., Tao M., Niu Y. (2015). *Microcystis aeruginosa/Pseudomonas pseudoalcaligenes* interaction effects on off-flavors in algae/bacteria co-culture system under different temperatures. J. Environ. Sci..

[49] Stengel D.B., Connan S., Popper Z.A. (2011). Algal chemodiversity and bioactivity: Sources of natural variability and implications for commercial application. Biotechnol. Adv..

[50] Thangam R., Suresh V., Asenath Princy W., Rajkumar M., Senthilkumar N., Gunasekaran P., Rengasamy R., Anbazhagan C., Kaveri K., Kannan S. (2013). C-Phycocyanin from *Oscillatoria tenuis* exhibited an antioxidant and in vitro antiproliferative activity through induction of apoptosis and G 0/G1 cell cycle arrest. Food Chem..

[51] Soria-Mercado I.E., Pereira A., Cao Z., Murray T.F., Gerwick W.H. (2009). Alotamide A, a novel neuropharmacological agent from the marine cyanobacterium *Lyngbya bouillonii*. Org. Lett..

[52] Medina-Gundrum L., Cerna C., Gomez L.R., Yochmowitz M., Weitman S. (2003). AMD473 (ZD0473) exhibits marked in vitro anticancer activity in human tumor specimens taken directly from patients. Anticancer. Drugs.

[53] Umemura K., Yanase K., Suzuki M., Okutani K., Yamori T., Andoh T. (2003). Inhibition of DNA topoisomerases I and II, and growth inhibition of human cancer cell lines by a marine microalgal polysaccharide. Biochem. Pharmacol..

[54] Samarakoon K.W., Ko J.Y., Lee J.H., Kwon O.N., Kim S.W., Jeon Y.J. (2014). Apoptotic anticancer activity of a novel fatty alcohol ester isolated from cultured marine diatom, *Phaeodactylum tricornutum*. J. Funct. Foods.

[55] Zorofchian Moghadamtousi S., Karimian H., Khanabdali R., Razavi M., Firoozinia M., Zandi K., Abdul Kadir H. (2014). Anticancer and antitumor potential of fucoidan and fucoxanthin, two main metabolites isolated from brown algae. Sci. World J..

[56] Usoltseva (Menshova) R.V., Anastyuk S.D., Shevchenko N.M., Zvyagintseva T.N., Ermakova S.P. (2016). The comparison of structure and anticancer activity in vitro of polysaccharides from brown algae Alaria marginata and A. angusta. Carbohydr. Polym..

[57] Itoh H., Noda H., Amano H., Ito H. (1995). Immunological analysis of inhibition of lung metastases by fucoidan (GIV-A) prepared from brown seaweed Sargassum thunbergii. Anticancer Res..

[58] Riou D., Colliec-Jouault S., Pinczon Du Sel D., Bôsch S., Siavoshian S., Le Bert V., Tomasoni C., Sinquin C., Durand P., Roussakis C. (1996). Antitumor and antiproliferative effects of a fucan extracted from ascophyllum nodosum against a non-small-cell bronchopulmonary carcinoma line. Anticancer Res..

[59] Aisa Y., Miyakawa Y., Nakazato T., Shibata H., Saito K., Ikeda Y., Kizaki M. (2005). Fucoidan induces apoptosis of human HS-Sultan cells accompanied by activation of caspase-3 and down-regulation of ERK pathways. Am. J. Hematol..

[60] Wang J., Zhang Q., Zhang Z., Song H., Li P. (2010). Potential antioxidant and anticoagulant capacity of low molecular weight fucoidan fractions extracted from Laminaria japonica. Int. J. Biol. Macromol..

[61] Yoon S.J., Pyun Y.R., Hwang J.K., Mourão P.A.S. (2007). A sulfated fucan from the brown alga Laminaria cichorioides has mainly heparin cofactor II-dependent anticoagulant activity. Carbohydr. Res..

[62] Atashrazm F., Lowenthal R.M., Woods G.M., Holloway A.F., Dickinson J.L. (2015). Fucoidan and cancer: A multifunctional molecule with anticancer potential. Mar. Drugs.

[63] Eriksen N.T. (2008). Production of phycocyanin-A pigment with applications in biology, biotechnology, foods and medicine. Appl. Microbiol. Biotechnol..

[64] Madamwar D., Kaushal A., Patel D.K., Desai S.N., Upadhyay K., Devkar R.V. (2015). Cyanobacterial phycoerythrin purified from marine Lyngbya sp. Induces apoptosis in lung carcinoma cells. Bangladesh J. Pharmacol..

[65] Hamann M.T., Scheuer P. (1993). Kahalalide F: A bioactive depsipeptide from the sacoglossan mollusk Elysia rufescens and the green alga Bryopsis sp.. J. Am. Chem. Soc..

[66] Hamann M.T. (2004). Technology evaluation: Kahalalide F, PharmaMar. Curr. Opin. Mol. Ther..

[67] López-Macià A., Jiménez J.C., Royo M., Giralt E., Albericio F. (2001). Synthesis and structure determination of kahalalide F (1,2). J. Am. Chem. Soc..

[68] Burja A.M., Banaigs B., Abou-Mansour E., Grant Burgess J., Wright P.C. (2001). Marine cyanobacteria—A prolific source of natural products. Tetrahedron.

[69] Taniguchi M., Nunnery J.K., Engene N., Esquenazi E., Byrum T., Dorrestein P.C., Gerwick W.H. (2010). Palmyramide a, a cyclic depsipeptide from a palmyra atoll collection of the marine cyanobacterium *lyngbya majuscula*. J. Nat. Prod..

[70] Mevers E., Liu W.T., Engene N., Mohimani H., Byrum T., Pevzner P.A., Dorrestein P.C., Spadafora C., Gerwick W.H. (2011). Cytotoxic veraguamides, alkynyl bromide-containing cyclic depsipeptides from the marine cyanobacterium cf. *Oscillatoria margaritifera*. J. Nat. Prod..

[71] Sasso S., Pohnert G., Lohr M., Mittag M., Hertweck C. (2012). Microalgae in the postgenomic era: A blooming reservoir for new natural products. FEMS Microbiol. Rev..

[72] Varela J.C., Pereira H., Vila M., León R. (2015). Production of carotenoids by microalgae: Achievements and challenges. Photosynth. Res..

[73] Bhosale P. (2004). Environmental and cultural stimulants in the production of carotenoids from microorganisms. Appl. Microbiol. Biotechnol..

[74] Fernández-Sevilla J.M., Acién Fernández F.G., Molina Grima E. (2010). Biotechnological production of lutein and its applications. Appl. Microbiol. Biotechnol..

[75] Ko J.C., Chen J.C., Wang T.J., Zheng H.Y., Chen W.C., Chang P.Y., Lin Y.W. (2016). Astaxanthin down-regulates Rad51 expression via inactivation of AKT kinase to enhance mitomycin C-induced cytotoxicity in human non-small cell lung cancer cells. Biochem. Pharmacol..

[76] Khan M.N.A., Choi J.S., Lee M.C., Kim E., Nam T.J., Fujii H., Hong Y.K. (2008). Anti-inflammatory activities of methanol extracts from various seaweed species. J. Environ. Biol..

[77] Panis G., Carreon J.R. (2016). Commercial astaxanthin production derived by green alga *Haematococcus pluvialis*: A microalgae process model and a techno-economic assessment all through production line. Algal Res..

[78] Serini S., Fasano E., Piccioni E., Cittadini A.R., Calviello G. (2011). Differential anticancer effects of purified EPA and DHA and possible mechanisms involved. Curr. Med. Chem..

[79] Mernitz H., Lian F., Smith D.E., Meydani S.N., Wang X.D. (2009). Fish oil supplementation inhibits NNK-induced lung carcinogenesis in the A/J mouse. Nutr. Cancer.

[80] Yao Q.-H., Zhang X.-C., Fu T., Gu J.-Z., Wang L., Wang Y., Lai Y.-B., Wang Y.-Q., Guo Y. (2014). ω-3 polyunsaturated fatty acids inhibit the proliferation of the lung adenocarcinoma cell line A549 in vitro. Mol. Med. Rep..

[81] Choi H., Hwang H., Chin J., Kim E., Lee J., Nam S.J., Lee B.C., Rho B.J., Kang H. (2011). Tuberatolides, potent FXR antagonists from the Korean marine tunicate *Botryllus tuberatus*. J. Nat. Prod..

[82] Kanadaswami C., Lee L.T., Lee P.P.H., Hwang J.J., Ke F.C., Huang Y.T., Lee M.T. (2005). The antitumor activities of flavonoids. In Vivo.

[83] Moosavi M.A., Yazdanparast R., Sanati M.H., Nejad A.S. (2005). 3-Hydrogenkwadaphnin targets inosine 5′-monophosphate dehydrogenase and triggers post-G1 arrest apoptosis in human leukemia cell lines. Int. J. Biochem. Cell Biol..

[84] Xiao B., Guo J., Liu D., Zhang S. (2007). Aloe-emodin induces in vitro G2/M arrest and alkaline phosphatase activation in human oral cancer KB cells. Oral Oncol..

[85] Lee Y.G., Lee K.W., Kim J.Y., Kim K.H., Lee H.J. (2004). Induction of apoptosis in a human lymphoma cell line by hydrophobic peptide fraction separated from anchovy sauce. Biofactors.

[86] Tzianabos A.O. (2000). Polysaccharide immunomodulators as therapeutic agents: Structural aspects and biologic function. Clin. Microbiol. Rev..

[87] Janmaat M.L., Rodriguez J.A., Jimeno J.M., Kruyt F.A.E., Giaccone G. (2005). Kahalalide F induces necrosis-like cell death that involves depletion of ErbB3 and inhibition of Akt signaling. Mol. Pharmacol..

[88] Gao J., Hamann M.T. (2011). Chemistry and biology of kahalalides. Chem. Rev..

[89] Cotter T.G. (2009). Apoptosis and cancer: The genesis of a research field. Nat. Rev. Cancer.

[90] Heimli H., Giske C., Naderi S., Drevon C.A., Hollung K. (2002). Eicosapentaenoic acid promotes apoptosis in Ramos cells via activation of caspase-3 and -9. Lipids.

[91] Rovito D., Giordano C., Vizza D., Plastina P., Barone I., Casaburi I., Lanzino M., De Amicis F., Sisci D., Mauro L. (2013). Omega-3 PUFA ethanolamides DHEA and EPEA induce autophagy through PPARγ activation in MCF-7 breast cancer cells. J. Cell. Physiol..

[92] Gravaghi C., La Perle K.M.D., Ogrodwski P., Kang J.X., Quimby F., Lipkin M., Lamprecht S.A. (2011). Cox-2 expression, PGE2and cytokines production are inhibited by endogenously synthesized n-3 PUFAs in inflamed colon of fat-1 mice. J. Nutr. Biochem..

[93] Liu G., Bibus D.M., Bode A.M., Ma W.Y., Holman R.T., Dong Z. (2001). Omega 3 but not omega 6 fatty acids inhibit AP-1 activity and cell transformation in JB6 cells. Proc. Natl. Acad. Sci. USA.

[94] Ben-Dor A., Nahum A., Danilenko M., Giat Y., Stahl W., Martin H.D., Emmerich T., Noy N., Levy J., Sharoni Y. (2001). Effects of acyclo-retinoic acid and lycopene on activation of the retinoic acid receptor and proliferation of mammary cancer cells. Arch. Biochem. Biophys..

[95] Judé S., Martel E., Vincent F., Besson P., Couet C., Ogilvie G.K., Pinault M., De Chalendar C., Bougnoux P., Richard S. (2007). Dietary long-chain n-3 fatty acids modify blood and cardiac phospholipids and reduce protein kinase-C-δ and protein kinase-C-ε translocation. Br. J. Nutr..

[96] Li B., Gao M.H., Chu X.M., Teng L., Lv C.Y., Yang P., Yin Q.F. (2015). The synergistic antitumor effects of all-trans retinoic acid and C-phycocyanin on the lung cancer A549 cells in vitro and in vivo. Eur. J. Pharmacol..

[97] Xu X., Zhang Y., Qu D., Liu H.B., Gu X., Jiao G.Y., Zhao L. (2013). Combined anticancer activity of osthole and cisplatin in NCI-H460 lung cancer cells in vitro. Exp. Ther. Med..

[98] Yang L., Wang P., Wang H., Li Q., Teng H., Liu Z., Yang W., Hou L., Zou X. (2013). Fucoidan derived from *Undaria pinnatifida* induces apoptosis in human hepatocellular carcinoma SMMC-7721 cells via the ROS-mediated mitochondrial pathway. Mar. Drugs.

[99] Zhou G., Sheng W., Yao W., Wang C. (2006). Effect of low molecular λ-carrageenan from *Chondrus ocellatus* on antitumor H-22 activity of 5-Fu. Pharmacol. Res..

[100] Lins K.O.A.L., Bezerra D.P., Alves A.P.N.N., Alencar N.M.N., Lima M.W., Torres V.M., Farias W.R., Pessoa C., de Moraes M.O., Costa-Lotufo L.V. (2009). Antitumor properties of a sulfated polysaccharide from the red seaweed *Champia feldmannii* (Diaz-Pifferer). J. Appl. Toxicol..

[101] Wang X., Zhang Z. (2014). The antitumor activity of a red alga polysaccharide complexes carrying 5-fluorouracil. Int. J. Biol. Macromol..

[102] Charkie J. (2014). Psammaplin A: A Putative Adjuvant for DNA Damaging Therapies. J. Cancer Sci. Ther..

[103] Abd El-Hack M.E., Abdelnour S., Alagawany M., Abdo M., Sakr M.A., Khafaga A.F., Mahgoub S.A., Elnesr S.S., Gebriel M.G. (2019). Microalgae in modern cancer therapy: Current knowledge. Biomed. Pharmacother..

[104] Khan M.I., Shin J.H., Kim J.D. (2018). The promising future of microalgae: Current status, challenges, and optimization of a sustainable and renewable industry for biofuels, feed, and other products. Microb. Cell Fact..

[105] Mata T.M., Martins A.A., Caetano N.S. (2010). Microalgae for biodiesel production and other applications: A review. Renew. Sustain. Energy Rev..

[106] Mulders K.J.M., Lamers P.P., Martens D.E., Wijffels R.H. (2014). Phototrophic pigment production with microalgae: Biological constraints and opportunities. J. Phycol..

[107] González L.E., Díaz G.C., Aranda D.A.G., Cruz Y.R., Fortes M.M. (2015). Biodiesel Production Based in Microalgae: A Biorefinery Approach. Nat. Sci..

[108] Tredici M.R., Rodolfi L., Biondi N., Bassi N., Sampietro G. (2016). Techno-economic analysis of microalgal biomass production in a 1-ha Green Wall Panel (GWP®) plant. Algal Res..

[109] Ummalyma S.B., Gnansounou E., Sukumaran R.K., Sindhu R., Pandey A., Sahoo D. (2017). Bioflocculation: An alternative strategy for harvesting of microalgae–An overview. Bioresour. Technol..

[110] Van Den Hende S., Vervaeren H., Desmet S., Boon N. (2011). Bioflocculation of microalgae and bacteria combined with flue gas to improve sewage treatment. New Biotechnol..

[111] Ndikubwimana T., Zeng X., Murwanashyaka T., Manirafasha E., He N., Shao W., Lu Y. (2016). Harvesting of freshwater microalgae with microbial bioflocculant: A pilot-scale study. Biotechnol. Biofuels.

[112] Alam A., Vandamme D., Chun W., Zhao X., Foubert I., Wang Z., Muylaert K., Yuan Z. (2016). Bioflocculation as an innovative harvesting strategy for microalgae. Rev. Environ. Sci. Biotechnol..

[113] Ventura S.P.M., Nobre B.P., Ertekin F., Hayes M., Garciá-Vaquero M., Vieira F., Koc M., Gouveia L., Aires-Barros M.R., Palavra A.M.F. (2017). Extraction of value-added compounds from microalgae. Microalgae-Based Biofuels and Bioproducts: From Feedstock Cultivation to End-Products.

[114] El-Sheekh M.M., El-Kassas H.Y. (2014). Application of biosynthesized silver nanoparticles against a cancer promoter cyanobacterium, *Microcystis aeruginosa*. Asian Pac. J. Cancer Prev..

